# Spondarthritis in the Triassic

**DOI:** 10.1371/journal.pone.0013425

**Published:** 2010-10-14

**Authors:** Juan Carlos Cisneros, Uiara Gomes Cabral, Frikkie de Beer, Ross Damiani, Daniel Costa Fortier

**Affiliations:** 1 Centro de Ciências da Natureza, Universidade Federal do Piauí, Teresina, Brazil; 2 Departamento de Geologia e Paleontologia, Museu Nacional, Universidade Federal do Rio de Janeiro, Rio de Janeiro, Brazil; 3 Nuclear Technology Division, South African Nuclear Energy Corporation, Pretoria, South Africa; 4 Staatliches Museum für Naturkunde Stuttgart, Stuttgart, Germany; 5 Departamento de Paleontologia e Estratigrafia, Universidade Federal do Rio Grande do Sul, Porto Alegre, Brazil; University of Padova, Italy

## Abstract

**Background:**

The evidence of several forms of arthritis has been well documented in the fossil record. However, for pre-Cenozoic vertebrates, especially regarding reptiles, this record is rather scarce. In this work we present a case report of spondarthritis found in a vertebral series that belonged to a carnivorous archosaurian reptile from the Lower Triassic (∼245 million years old) of the South African Karoo.

**Methodology/Principal Findings:**

Neutron tomography confirmed macroscopic data, revealing the ossification of the entire intervertebral disc space (both annulus fibrosus and nucleus pulposus), which supports the diagnosis of spondarthritis.

**Conclusions/Significance:**

The presence of spondarthritis in the new specimen represents by far the earliest evidence of any form of arthritis in the fossil record. The present find is nearly 100 million years older than the previous oldest report of this pathology, based on a Late Jurassic dinosaur. Spondarthritis may have indirectly contributed to the death of the animal under study.

## Introduction

The Karoo Basin, comprising South Africa and neighboring countries, has produced an unparalleled wealth of past life, being notable for its detailed record of fossil vertebrates that highlight the Permo-Triassic biotic crisis [1]. In this contribution, we analyze the vertebral remains of a carnivorous reptile from the Lower Triassic of South Africa, a specimen that shows macroscopic signs of a severe bone pathology. The condition is here identified as spondarthritis, which encompasses a diverse group of related inflammatory arthritides that share multiple clinical features as well as common genetic predisposing factors [2], and it represents the oldest instance of this pathology hitherto known.

## Materials and Methods

**Figure 1:**
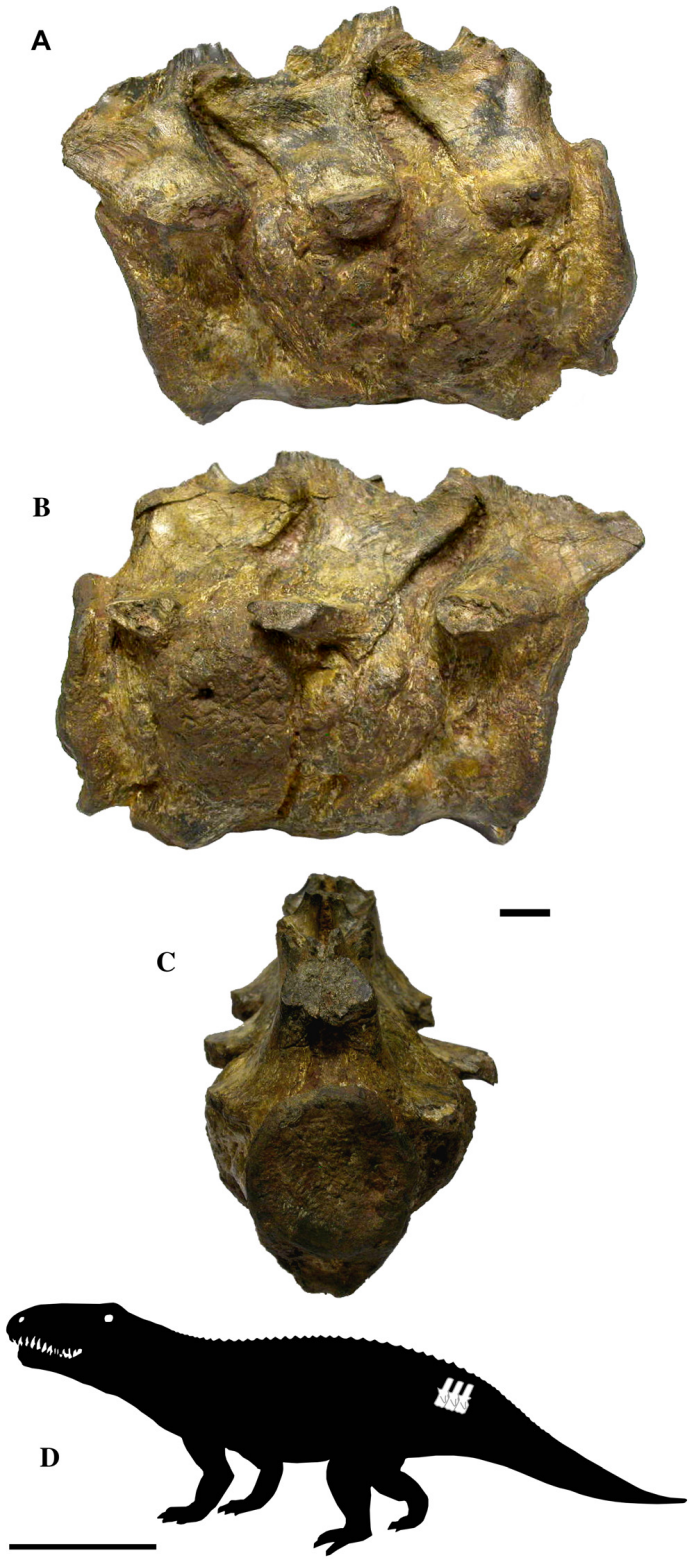
External views of vertebrae BP-1-5796. A. Left lateral view. B. Right lateral view. C. Posterior view. D. Schematic drawing of a basal archosaurian reptile, showing the likely placement of the vertebral series within the caudal region of the column. Scale bars represent 10 mm for (A–B), and 1 m for (D).

Specimen BP-1-5796 (stored at the Bernard Price Institute for Palaeontological Research, Johannesburg) consists of three articulated anterior caudal vertebrae of a large, basal archosaurian reptile (Figure 1). The specimen was collected at Driefontein District, Free State Province, South Africa. This locality produces a fauna that is considered to represent the lower portion of the *Cynognathus* Zone (*Cynognathus* subzone A), of late Early Triassic age (Olenekian, ∼245 Ma) [3], [4], [5].

Neutron tomography, a nondestructive technique, was employed in order to examine the internal structure of the specimen. This was performed through the SANRAD tomography facility at the SAFARI-1 nuclear research reactor operated by the South African Nuclear Energy Corporation (NECSA) in Pretoria. The reactor has a design power of 20MW and provides a neutron flux of 1.2×107 n.cm-2.s-1 at beam port no-2 at the object under investigation. For detailed specifications of the SANRAD tomography facility see [6], [7]. The specimen was placed on top of a rotating desk, on which a total of 180 projections in 180 angular degrees were made (Video S1). The image reconstruction procedure was performed on IDL based GSECARS Tomography Processing Software [8]. VGStudio MAX 2.1 software from Volume-Graphics was used for 3D rendering, segmentation and slicing of the images obtained.

## Results

The specimen here studied is composed by three nearly complete, fused vertebral bodies and partial zygapophyses (Figure 1). The vertebrae are almost complete, except for the missing distal portions of the neural spines. Judging from the size of these vertebrae, it is assumed that they belonged to a large, probably old individual. These are overall well preserved and show no signs of taphonomical alteration. As the vertebrae are fused, it is not possible to provide the length of each vertebra, the total length of the specimen being 116 mm.

**Figure 2:**
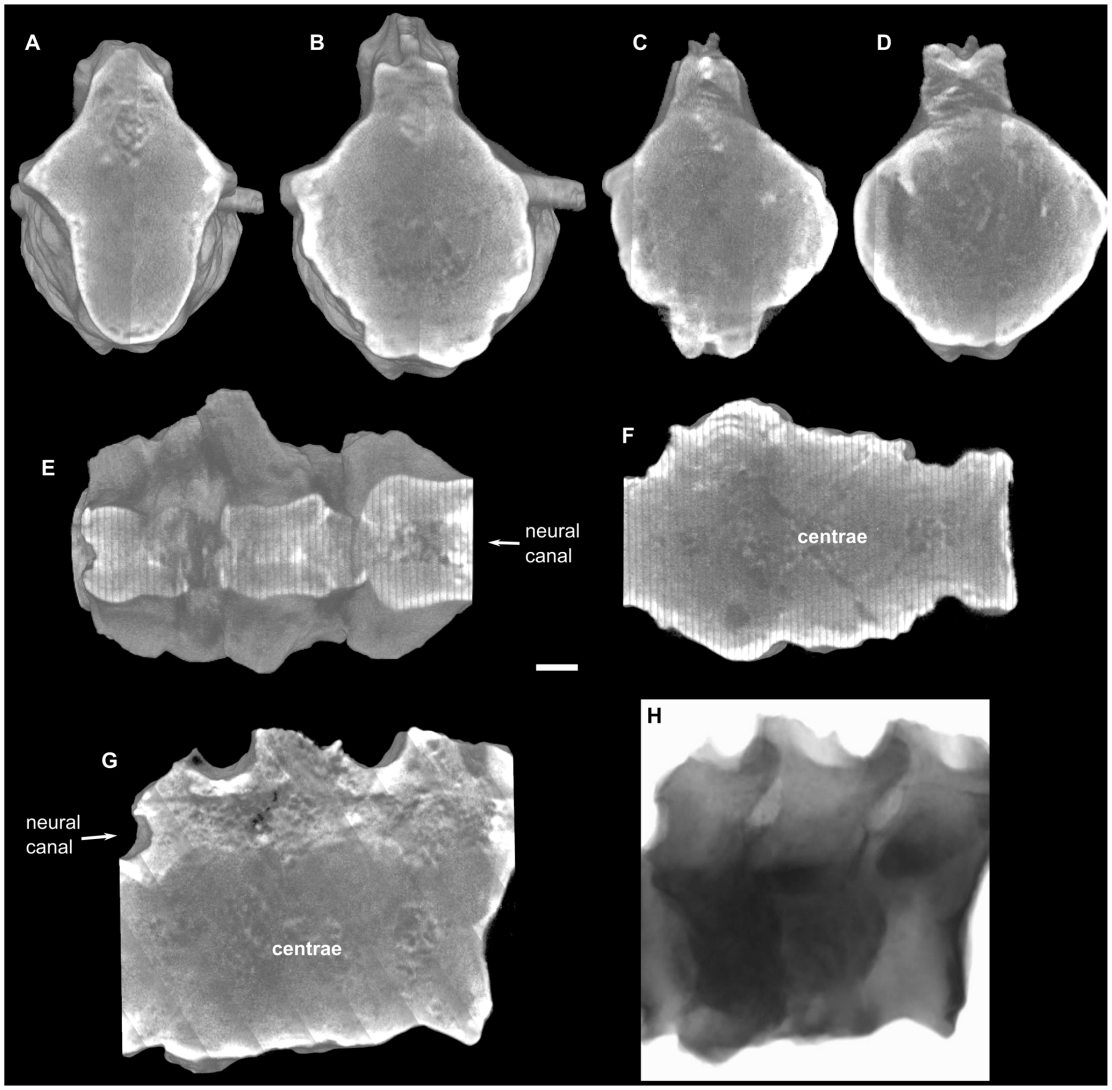
Neutron tomographies of vertebrae BP-1-5796. A. First (anterior to posterior direction) preserved vertebrae. B. Second vertebrae. C–D. Third vertebrae. A–C. Transversal slices, in anterior view, at level of anterior border of the transverse process. D. Transversal slice, in anterior view, at level of maximum thickness of inflammation. E–F. Coronal slices of the vertebral series in dorsal view, (E) at level of the neural canal, (F) at level of the centrae, anterior to the left. G. Sagittal slice of the vertebral series in right view. H. Raw neutron image in right lateral view. Scale bar represents 10 mm.

The osseous reactions in all three vertebrae are very similar, however, the two posterior vertebrae are visibly more affected (Figures 1A–C and 2A–D). In these vertebrae, the lateral and ventral surfaces of the vertebral bodies exhibit an exuberant osseous overgrowth, being located below the left and right transverse processes. This osseous growth precludes the visualization of intervertebral limits. Neither zygapophyses or neural arcs were affected, due to this, they are not fused, and the space between these structures is filled with sediment.

The neutron tomography (Figure 2) revealed no traces of fracture or trauma in the vertebral series. Through the tomography we observed that the intervertebral disc spaces (both annulus fibrosus and nucleus pulposus) are totally ossified, producing the complete ankylosis of the vertebrae (Figure 2F and G, Video S2 and S4). In this way, it is not possible to distinguish the limits of each vertebral element. No evidence of zygapophyseal ankylosis, however, was found through the tomography (Figure 2E and H, Video S1, S3 and S4), thus, confirming what was recognized by the external exam of the specimen. Neutron images also showed that in the innermost layer of the vertebral bodies the trabecular bone forms trabecular bridges that follow a regular pattern across the ankylosed areas (Figure 2G, Video S4). The absence of an irregular pattern of trabecular bone allows to discard the hypothesis of an infectious process or tumor [9], [10].

### Differential diagnosis

There are developmental anomalies that can lead to similar vertebral ankyloses to the one observed in the vertebrae here studied. This is the case of congenital vertebral synostosis, which can occur both by an alteration of the secondary embrionary segmentation or due to absence of intervertebral discs [11]. It is not common, however, that bones affected solely by this anomaly bear an external osseous protuberance such as the one found in our specimen.

Regarding non-congenital pathologies, there is a variety of processes that can affect, directly or indirectly, leading to vertebral ankylosis. Fractures on articular surfaces may result in the damage and osseous ankylosis of these articulations. This problem is related to cominutive fractures, where more than one articular surface is involved with the fibrocartilage callus, fusing two or more bones [12]. In vertebrae, ankylosis is a common sequel to fractures by compression of one or more vertebral bodies, this occurs through a mechanism of fibrocartilage callus production that extends to one or more adjacent vertebrae [12]. In cases of ankylosis resulting from trauma, evidence of one or more fracture lines often remains, allowing recognition of this process. However, there is no evidence that the ankylosis in the vertebrae here studied was the result of a traumatic process. There is neither presence of immature bone nor evidence of fracture lines.

Rheumatoid arthritis of the vertebral column, an illness that may also lead to vertebral ankylosis, is characterized by progressive synostosis of the following elements: anterior and posterior ligaments, capsules and small intervertebral articulations, and intervertebral ligaments [11]. However, intervertebral discs are not affected, being a pathological condition that is contradicted by the specimen under study. Furthermore, the bone reaction in rheumatoid arthritis is minimal or absent [13].

Another disorder that can produce vertebral ankylosis is the diffuse idiopathic skeletal hyperostosis (DISH). DISH describes a phenomenon characterized by calcification and ossification of entheseal sites. The ossification and calcification of the anterolateral aspect of the thoracic spine are regarded as a hallmark of the disease, however, it is not limited to the spine, as it has often been reported to involve peripheral sites as well [14]. It is important to note that DISH should not be considered merely as an isolated spinal condition but rather a systemic disease. DISH should be regarded as an extensive proliferative musculoskeletal disease with clinical and metabolic derangements [14]. Resnick and Niwayama [15] defined the classification criterion that is currently used: involvement of at least four contiguous thoracic vertebral segments, preservation of intervertebral disc spaces, and the absence of apophyseal joint degeneration or sacroiliac inflammatory changes. The condition is recognized radiographically by the presence of “flowing” ossification along the anterolateral margins of the vertebrae and the absence of changes of spondyloarthropathy or degenerative spondylosis [16].

Osteoarthritis is a non-erosive type of arthritis, in which the primary sites of tissue injury are the cartilage of the joint and the subchondral bone, directly underlying and supporting it [13]. In osteoarthritis, overgrowth of bone (osteophytes) do occur, but not bone resorption. Vertebral phenomena, however, are usually not a result of osteoarthritis. The term osteoarthritis is properly restricted in application to diarthrodial joints, thus, excluding the disk spaces [17]. The only form of spine disease accurately referred to as osteoarthritis is that producing osseous overgrowth (osteophytes) of the zygapophyseal joints [10].

The term “spondyloarthropathy” is still frequently employed in the literature (both medical and paleopathological), however, it is recommended the use of the term “spondarthritis” (or “spondyloarthritis”), because the suffix “arthropathy” is too ambiguous and does not indicate the inflammatory nature of the condition [18], [19], [20]. The spondarthrites are characterized by axial and peripheral arthritis, absence of rheumatoid factor, increased frequency of HLA-B27, overlapping syndromes, familial aggregation, and mutual association within patients and families [20]. The following diagnostic criteria have been employed in skeletal analyses: evidence of zygapophyseal or sacroiliac joint erosion or ankylosis; asymmetrical arthritis patterns; reactive new bone formation; syndesmophytes (being either marginals or calcifications/ossifications within the annulus fibrosus); or, peripheral joint ankylosis [17], [21]. Zygapophyseal joint erosion or ankylosis and ossification within annulus fibrosus are pathognomonic signals [17]. In addition, Golding [22] reports that a more florid type of periosteal reaction occurs in the spondarthritis group of arthritic disorders, such as Reiter's disease and psoriatic arthritis. Furthermore, the presence of annulus fibrosus ossification in combination with typical syndesmophytes, allows to discard the diagnosis for DISH, discarthrosis and infectious spondylitis [19].

The diagnosis for some diseases is defined based on the presence of characteristic features in certain bones or in a group of them. For this reason, the study of pathologies on isolated bones may become a delicate question. However, despite of the obstacles that are inherent to diagnosing isolated bones, these instances of pathologies do have their own value, specially in relation to behavior and habitat. In perspective, they reinforce through understanding the population that they represent [17]. Through the differential diagnosis of the features found in the vertebral series, it is possible to discard in our specimen the possibility of congenital anomaly, fracture, tumor, rheumatoid arthritis, DISH, or osteoarthritis. Based on the present analysis, the most probable explanation, supported both by the elimination of other hypothesis and by the observed characteristics, is that the vertebral ankylosis was generated by a process of spondarthritis.

## Discussion

It is not always feasible to identify the variety of spondarthritis recorded. Not even clinically, when it is possible to actually talk with an afflicted human. The presence of ancillary body system involvement (e.g., dermatologic, genito-urinary symptom) allows spondarthrites to be distinguished clinically, but discrimination among them may not be possible when only the skeleton is available for analysis [13].

The present find constitutes the oldest instance of spondarthritis in the fossil record. The previous oldest evidence of this pathology was found on vertebrae from the dinosaur *Camarasaurus* from Utah, USA [23] of latest Jurassic age (Tithonian, ∼147 Ma). Thus, our find extends the presence of spondarthritis in the fossil record by nearly one hundred million years, back to the Early Triassic.

The vertebrae under study constitute the remnants of a large, and presumably old, carnivorous reptile. Being affected by this pathology, the individual would suffer an increasing constraint of the movements of its axial skeleton that slowly would hamper its faculty of locomotion. Such condition was surely disadvantageous in a number of activities that may require great physical effort, such as praying and territory defense. By gradually imposing restrictions in activities that are vital to the individual (i.e. acquisition of food items) the development of spondarthritis may have indirectly contributed to the death of this individual.

### Conclusions

A severe pathology was recognized in a caudal vertebral series of a basal archosaurian reptile from the Lower Triassic of the South African Karoo. Both macroscopic examination and neutron tomography data revealed features that are diagnostic of spondarthritis. As such, the present find represents the earliest example of this pathology in the fossil record. The disease was likely an indirect cause of the death of the animal.

## Supporting Information

Video S1 Rotation of vertebrae BP-1-5796, made from 180 neutron images in 180 angular degrees.
(3.13 MB MPG)

Video S2 Parasagittal slices of vertebrae BP-1-5796, from left to right.
(1.04 MB MP4)

Video S3 Transversal slices of vertebrae BP-1-5796, in posterior to anterior direction.
(2.21 MB MP4)

Video S4 Coronal slices of vertebrae BP-1-5796, in ventral to dorsal direction.
(1.17 MB MP4)
